# Risk factors and mortality of patients undergoing hip fracture surgery: a one-year follow-up study

**DOI:** 10.1038/s41598-020-66614-5

**Published:** 2020-06-15

**Authors:** Pierre Huette, Osama Abou-Arab, Az-Eddine Djebara, Benjamin Terrasi, Christophe Beyls, Pierre-Grégoire Guinot, Eric Havet, Hervé Dupont, Emmanuel Lorne, Alexandre Ntouba, Yazine Mahjoub

**Affiliations:** 1 Department of Anaesthesiology and Critical Care Medicine. Amiens University Hospital. F- 80054, Amiens, France; 2 Department of orthopedic surgery. Amiens University Hospital. F- 80054, Amiens, France; 3 Department of Anaesthesiology and Critical Care Medicine. Dijon University Hospital. F- 21000, Dijon, France

**Keywords:** Trauma, Risk factors, Geriatrics, Disease-free survival

## Abstract

Hip fracture (HF) remains a main issue in the elderly patient. About 1.6 million patients a year worldwide are victims of a HF. Their incidence is expected to rise with the aging of the world’s population. Identifying risk factors is mandatory in order to reduce mortality and morbidity. The aim of the study was to identify risk factors of 1-year mortality after HF surgery. We performed an observational, prospective, single-center study at Amiens University Hospital (Amiens, France). After ethical approval, we consecutively included all patients with a HF who underwent surgery between June 2016 and June 2017. Perioperative data were collected from medical charts and by interviews. Mortality rate at 12 months was recorded. Univariate analysis was performed and mortality risk factors were investigated using a Cox model. 309 patients were analyzed during this follow-up. Mortality at 1 year was 23.9%. Time to surgery over 48 hours involved 181 patients (58.6%) while 128 patients (41.4%) had surgery within the 48 hours following the hospital admission. Independent factors associated with 1-year mortality were: age (HR at 1.059 (95%CI [1.005–1.116], p = 0,032), Lee score ≥ 3 (HR at 1,52 (95% CI [1,052–2,198], p = 0.026) and time to surgery over 48 hours (HR of 1.057 (95% CI [1.007–1.108], p = 0.024). Age, delayed surgical (over 48 hours) management and medical history are important risk factors of 1-year mortality in this French cohort

## Introduction

Hip fracture (HF) remains a main issue in the elderly patient. As bone loss tends to decreases in association with osteoporosis and osteopenia, the risk of hip fracture increases^1^. HF represents a health care concern as the incidence and the mortality rate remains high^2,3^. The world estimation of patients with HF is around 1.6 million a year^4^. In France, 150 000 patients each year suffer HF. The mortality rate varies from 8 to 36% depending on the country^5–7^. Only 50% patients will have independent living preservation following HF management^8^. Recognized risk factors for HF are old age, reduced activity, female gender and osteoporosis. Though, in every day anesthesia practice, HF represents a surgery all practitioners have to deal with. However, data on perioperative management are lacking or rely on small sample size. Evidence is of low quality or even non-existent.

Besides HF management mainly depends on each country healthcare system. Literature is abundant on national observational cohort^9,10^. However, data are rarely comparable from one country to another depending on its healthcare system. Moreover, the management requires a multidisciplinary approach including geriatrician, surgeon and anesthetist. It seems that a care organization within a dedicated orthogeriatric ward can reduce long-term mortality^11–13^.

A recent meta-analysis revealed residential status, pulmonary disease, cardiovascular disease, diabetes, and time to surgery increased the risk of mortality after HF surgery^14^.

It also seems that surgery delay counts for the poor prognosis as reported in another meta-analysis of 190000 patients^15^. That finding is controversial as reported as non-significant in others studies^16^.

To date, there is no study in France that evaluated mortality and risk factors after HF during the perioperative time. The aim of the study was to determine the mortality rate at 1-year in a French cohort and to identify mortality risk factors, especially preventable risk factors.

## Methods

### Ethics

We performed an observational, prospective, single-center study at Amiens University Hospital (Amiens, France). Ethical approval for this study (Ethical Committee N° RNI2016–04 the 26^th^ of may 2016) was provided by the “*Comité de Protection des Personnes Nord-Ouest II”* of Amiens University Hospital, Amiens, France. The trial was registered on ClinicalTrials.gov (identifier: NCT03117868). The study complied with the Declaration of Helsinki on ethical principles for medical research involving human subjects. Written informed consent was given by all participating patients or their legal representatives and written informed consent was obtained from the next of kin in case of data used from dead patients. The present report was drafted in line with the STROBE statement for observational studies in epidemiology^17^.

### Study population

Patients with HF admitted to the traumatology department between June 2016 and June 2017 were prospectively enrolled in the study. The inclusion criteria were age over 65 years, surgical managing of traumatic HF, and agreement to participate to the study. For patients with cognitive impairment, the closest relative or caregiver was informed. The non-inclusion criteria were non-traumatic HF (pathological fracture), open fracture, traffic accident and periprosthetic fracture.

### Measures and follow-up

Patient data were prospectively collected over a 1-year period. The following data were collected from medical records or interviews: age, gender, weight, body mass index (BMI), medical history (dyslipidemia, hypertension, diabetes, active smoking) and drug at baseline, ASA status as defined by the American Society of Anesthesiologists anesthesia and the LEE score^18^, pre-fracture functional level was assessed with the ADL (activity of daily living) score graded from 1 to 6 (full independence: the ability to do all 6 ADL without assistance; partial dependence as the ability to do 4 or 5 ADL without assistance; and total dependence as the ability to do 3 ADL or fewer without assistance), residential status (1:own home; 2: nursing home). The following biologic test results were collected: preoperative albumin, hemoglobin level at hospital admission, hemoglobin level the day before surgery and hemoglobin level on 1^st^ and 3^th^ postoperative days.

The following surgical data were collected: hip fracture type (total hip arthroplasty, hemiarthroplasty, dynamic hip screw with plate or intra medullar hip screw), time to surgery (≤ 48 h or >48 h, dating from the hospital admission to the beginning of surgery), anesthesia type (general or regional anesthesia (neuraxial technique, peripheral nerve block)) and the hospital length of stay (days). The time to surgery was defined by the delay between hospital admission and start of the surgery. Medical or surgical perioperative complications were recorded. Acute kidney injury (AKI) was defined as serum creatinine >1.5 baseline or increase >26.5 mmol/l.

### Mortality data collection

Each patient was followed up with an orthopaedic surgery consultation organised at 1 month, 3 months, 6 months and 1 year from the fracture. When patient did not come for consultation, the status dead or alive at each time (1,3,6 and 12 months) was followed up by calling the attending physician, the patient, the trustworthy person or the city hall to request the death registry.

### Statistical analysis

Data were expressed as mean ± standard deviation (SD) if data were normally distributed or as median [interquartile range (IQR)] if not. Categorial data were expressed in numbers (percentage). Normal distribution was verified using Shapiro-Wilk test. Variables were compared in a Student’s t test, a Wilcoxon-Mann-Whitney test, a chi-squared test or a Fischer exact test, as appropriate. Univariate analysis was used to identify risk factors of death at 12 months. Cox proportional hazards were used to display the adjusted cumulative hazard of the mortality at 12 months when p < 0.05 in univariate analysis. Time-to-event analyses were performed with the use of Kaplan-Meier estimates. Statistical analyses were performed with SPSS software for Mac (version 21, IBM, SPSS). The threshold for statistical significance was set to p < 0.05.

## Results

### Demographic data (Table 1)

**Figure 1:**
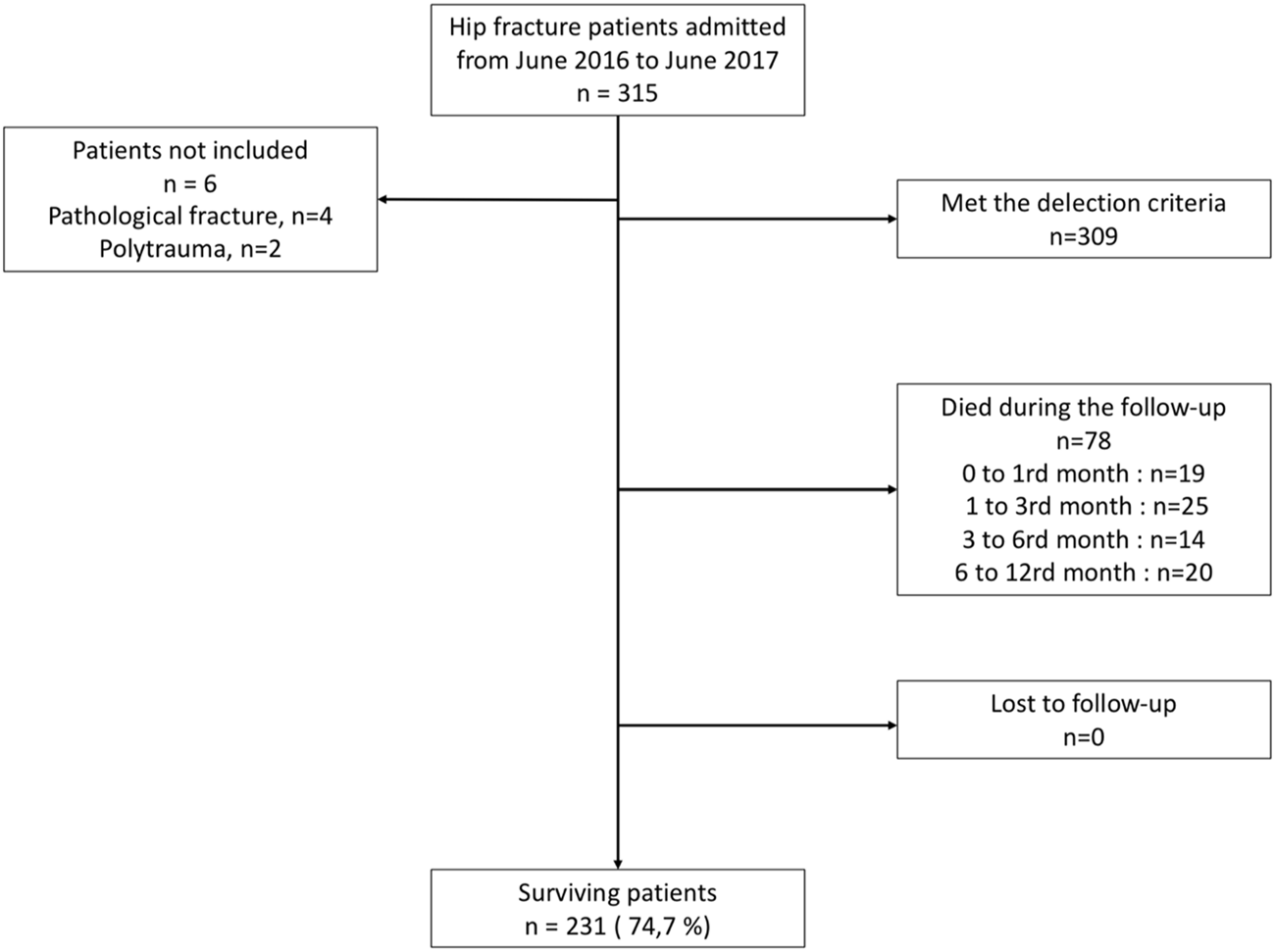
Flow chart.

A full description of the study cohort is provided in Table 1. We enrolled patients from June 2016 to June 2017 (Fig. 1). A total of 309 patients were included during the 1-year study period. No patient was excluded. No patient was lost to follow-up.

**Table 1:** Demographic data. Data are expressed as median [interquartile space] or numbers (percentage). **BMI:** body mass index; **ASA:** American Status Anaesthetist.

Variables	Overall population (n = 309)
Age (years)	85 [79–88]
Male gender (n; %)	82 (27)
BMI (kg m^−2^)	25 [22–27]
Albumin level (g l^−1^)	
<20	16 (5)
20–30	126 (41)
>30	130 42)
missing data	37 (12)
Comorbidities (n; %)	
Diabetes	54 (18)
Hypertension	220 (71)
Dyslipidaemia	92 (30)
Residential status (n; %)	
Own home	215 (69)
Nursing home	94 (31)
Pre-fracture functional status (n; %)	
*Full independence*	39 (13)
*Partial dependence*	37 (12)
*Total dependence*	84 (27)
*Missing data*	149 (48)
ASA status (n; %)	
1	9 (3)
2	91 (29)
3	192 (62)
4	17 (6)
Lee score (n; %)	
0	162 (52)
1	90 (29)
2	41 (13)
3	16 (5)

### Overall mortality rate

The mortality rate was 23.9% at one year following HF surgery. The mortality rate was respectively 6.1, 14.2 and 18.8% at respectively 1, 3, 6 months (p < 0.0001).

**Table 2:** Characteristics and univariate analysis of factors influencing 1-year mortality after hip fracture surgery. Data are expressed as median [interquartile space] or numbers (proportions). ADL, Activity of Daily Living; ASA, American Society of Anesthesiologists; BMI, Body Mass Index; AKI, Acute Kidney Injury.

	Alive (n = 231)	Dead (n = 78)	P value
Male gender (n; %)	57 (25)	25 (32)	0.202
Age (year)			
<65	26 (11)	3 (4)	0.001
66–75	44 (19)	5 (6)	
76–85	76 (33)	24 (31)	
>85	85 (37)	46 (59)	
BMI (kg m^−2^)	23.9 [21.1–27.0]	22.5 [21.1–27.0]	0.033
Albumin level (g l^−1^)			
*<20*	12 (5)	4 (5)	0.604
*20–*30	89 (38)	37 (47)	
> *30*	99 (44)	31 (40)	
*Missing data*	31 (13)	6 (8)	
Pre-fracture functional status			
*Full independence*	24 (10)	15 (19)	<0.001
*Partial dependence*	25 (11)	12 (15)	
*Total dependence*	77 (34)	7 (9)	
*Missing data*	105 (45)	54 (69)	
ASA status			
1	8 (3)	0 (0)	<0.001
2	81 (35)	10 (13)	
3	133 (58)	59 (76)	
4	9 (4)	8 (11)	
Lee			
0	136 (59)	26 (33)	<0.001
1	62(27)	28 (36)	
2	27 (12)	14 (18)	
3	6 (2)	10 (13)	
Surgery time (minutes)	54 [41–65]	47 [36–60]	0.035
Type of anaesthesia			
*General anaesthesia*	118 (51)	38 (49)	0.744
*Regional*	28 (13)	12(16)	
*Combined*	84 (36)	27 (35)	
Prosthesis type (n; %)			
*Total hip arthroplasty*	38 (16)	1(1)	0.003
*Hemiarthroplasty*	78 (34)	38 (49)	
*Dynamic hip screw*	66 (29)	19 (25)	
*Gamma nail*	45 (19)	16 (21)	
*Others*	4 (2)	3 (4)	
Residential status (n; %)			
Own home	173 (75)	42 (54)	<0.001
Nursing home	58 (25)	36 (46)	
Blood cell transfusion (n; %)	59 (25)	27 (35)	0.232
Time to surgery (n; %)			
*≤ 48 hours*	105 (45)	23 (29)	0.009
> *48 hours*	126 (55)	55 (71)	
Hospital discharge (days)	8 [6–10]	8.5 [6.2–12]	0.004
Perioperative complication (n; %)	50 (22)	20 (26)	0.280
AKI (Serum creatinine>1.5 baseline or increase>26.5 mmol/l) (n; %)	9 (4)	1 (1)	0.280

### Univariate analysis between survivor and dead *patients* (Table 2)

 At 1-year follow-up, mortality was significantly higher in patients living in nursing home (46% versus 25% in survivors, p < 0.01), in those with a ASA score at 3 (76% versus 58% in survivors, p < 0.01) and with a Lee score ≥ 3 (13% versus 2% in survivors, p < 0.01). The hemiarthroplasty is associated with higher mortality (49% versus 34% in survivors, p = 0.003). Time to surgery> 48 hours involved 181 patients (58,6%) while 128 patients (41,4%) had surgery within 48 hours of admission. There is a significant association between 1-year mortality and time to surgery, 55 patients (71%) died when surgery occurred after 48 hours whereas 23 (29%) died when operated within 48 hours (p = 0.009). Albumin preoperative level was not associated with 12-month mortality but the risk of mortality was higher in patients having a lower BMI (22.5 [21.1–27.0] versus 23.9 [21.1–27.0] in survivors, p = 0.033]. Furthermore, age was associated with 12-month mortality. We found no association between mortality at 1 year and length of stay, blood cell transfusion or the occurrence of perioperative medical or surgical complication. No association between post-operative AKI and mortality was found. In addition, there was no association between 1-year mortality and type of anesthesia.

Delaying surgery over 48 hours had several causes: 21% for antithrombotic drug management, 38% in high risk patients for additional test (echocardiography for example) and 41% for operating ranges issues. Clinical characteristics according to time of surgery and divided in subgroups according to the cause of delay are presented in the supplementary file. One-year mortality hazard ratio for Lee Score Criteria following surgical hip fracture management are presented in the supplementary file.

**Table 3:** Independent risk factors associated with 1-year mortality following surgical hip fracture management. HR: hazard ratio; P value using Cox model. BMI: Body Mass index.

**Variables**	HR	CI 95% HR	P value
Age	1,059	1.005–1.116	0.032
BMI	0.989	0.923 et 1.058	0.741
Prefracture status	0.737	0.540–1.005	0.054
Type of surgery	0.943	0.704, 1.263	0.693
ASA	0.744	0.392–1.413	0.367
Time to surgery> 48 h	1.057	1.007–1.108	0.024
Lee ≥ 3	1.52	1.052–2.198	0.026

### Cox model for prediction of one-year mortality and survival analysis by time to surgery and Lee score (Table 3)

**Figure 2:**
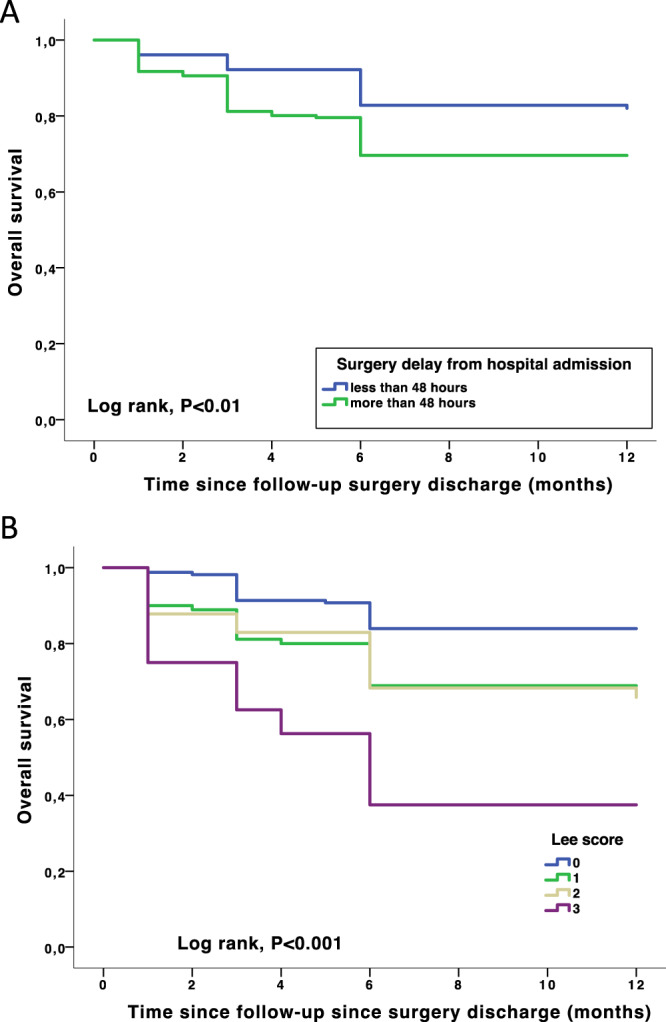
Time to death from surgery adjusted to delayed surgery (panel A) and Lee score (panel B). Lee score < 2: hazard ratio = 0.278; CI 95% = 0.128–0.606; P value = 0.001 Surgery discharge < 48 hours from hospital admission: hazard ratio = 0.640; CI 95% = 0.389–0.894; P value = 0.045.

Using a Cox model, 1-year mortality rate was increased when Lee score is ≥ 3 (HR at 1,52 (95% CI [1,052–2,198], p = 0.026), time to surgery> to 48 hours from hospital admission (HR of 1.057 (95% CI [1.007–1.108], p = 0.024), age was predictive (HR at 1.059 (95%CI [1.005–1.116]; p = 0,032). The Kaplan-Meier survival chart for time to surgery and Lee score is shown in Fig. 2.

## Discussion

In this study performed in a tertiary French hospital, the 1-year mortality rate for patient over 65 admitted for hip fracture is 23.9%. Age, time to surgery >48 h and Lee Score ≥3 were independent 1-year mortality risk factors.

Populations characteristics are similar to prior studies. The average age of patients following HF is 83.2 years, with a majority of ASA 2 or 3 status patients and a large proportion of women^4,19^. The mortality rate of 29% is 1-year mortality rate found in other studies^20^.

In our study we found an association between ASA score and Lee Score with mortality after HF surgery. The association between cardiovascular disease and mortality after HF surgery was highlighted by other studies^21–23^. In the literature, as in our study, the ASA score is associated with excess mortality in HF patients^24,25^. The ASA classification is an extremely common standard measure that identifies high-risks patients before surgery. This score enables a standardized risk assessment score for intra operative and post-operative risk^26,27^. A meta-analysis of Chang *et al*. conducted in 2018, including 25,349 patients from16 studies (13 prospective and 3 retrospective), found a significant association between mortality after HF and several risk factors including cardiac disease, diabetes and cancer^14^. Charleston Comorbidity Index (CCI) was described as able to predict postoperative complications after HF surgery however the odds ratios were not very large^28^. We did not assessed the CCI in the present study.

In accordance with previous studies, a time to surgery < 48 hours significantly increased the risk of death after HF surgery^29,30^. In our study, a time to surgery >48 h involved 181 patients (58.6%) while 128 patients (41.4%) had surgery within 48 hours of admission. Several hypotheses have been put forward to explain the existence of physiopathological processes specific to the HF. Among these, inflammation, hypercoagulability state, hypercatabolic state and stress^31,32^, are able to decompensate some comorbidities. Based on retrospective studies and European studies^15^ (no one was conducted in France), recent French guidelines (SFAR) recommended that hip surgery should be performed within 48 h after HF in order to increase survival rate^33^. French guidelines also supports orthogeriatric collaboration to improve mortality after hip repair. In March 2018, Chang *et al*. described a statistically significant association between increased “time-to-surgery” (>2 days vs <2 days; OR 1.91, 95% CI 1.14–3.18, p = 0.013) and mortality. Although it is clear that at this moment in time, the evidence remains conflicting. Our work is, to our knowledge, the first French prospective study on the field and emphasize the need to decrease the delay between HF and surgery. In our study, delaying surgery over 48 hours had several causes. 21% for antithrombotic drug management, 38% in high risk patients for additional test (echocardiography for example) but also operating range issues (41%). Even if further assessment for high risk patients seems reasonable, the benefit of early surgery for these patients should not be forgotten. Concerning antithrombotic drugs management, a retrospective work by Purushothaman *et al*. concluded that there is no increased risk of bleeding with clopidogrel for HF surgery^34^. Moreover, Mas-Atance J *et al*. have shown in a prospective study, that early surgery for patients receiving antiplatelet therapy reduced hospital length of stay without worsening clinical outcome.^35^.

In univariate, we identified nursing home residence as a risk factor for mortality following HF surgery. This result is in accordance with previous studies. Harris and al. so found that nursing home residence increased the risk of death after HF surgery in Australia^36^. In their meta-analysis, Chang and al. reported that patients living in a nursing home have a higher mortality than those at home^14^.

Using regional or general anesthesia has no influence on 1-year mortality in our follow-up study. Several studies compared the effects of regional anesthesia vs general anesthesia and found no difference on morbidity, mortality and hospitalization costs^13,37^. Our results are consistent with these recent data.

A recent study found that transfusion is associated with mortality for HF patients^25^. In our study, 27,8% of the patients were transfused, but we didn’t find any association between transfusion and 1-year mortality. The perioperative transfusion strategy has been the subject of numerous studies and some authors have investigated different strategies to reduce blood loss in order to avoid transfusion without increasing the risk of death^38,39^.

Giusti *et al*. showed that a multidisciplinary care model is superior to standard care^40^. Prestmo and *et al.*^41^ conducted a single-center, randomized trial and concluded that treatment of older patients with HF should be organized as orthogeriatric care. The absence of a dedicated orthogeriatric ward could explain the surgical delay in our medical center.

Our study presents some limits. First, as it is a single-center, observational study, some unknown confounding factors may exist. Secondly, we did not report data on anesthesia management. It is well known that ventilation, anesthesia depth and hemodynamic management may have a significant impact on postoperative outcomes and mortality rate.

The methodological strengths of our study include its prospective design and the long-term (1year) follow- up period. To our knowledge, no previous study evaluated risk factors of long term mortality in France. Moreover, as we had a very low rate of missing data, our sample can be considered as representative of the population of the region.

## Conclusion

In this first prospective study on long-term mortality in France, we found a 1-year mortality rate of 23.9%. Age, time to surgery >48 h and Lee score ≥3 are independent factor of 1-year mortality following HF surgery. Early surgical management of patients suffering from HF remains an important mean to reduce mortality.

## Supplementary information

Supplementary Information.
