# Supplementary material for: Risk factors and mortality of patients undergoing hip fracture surgery: a one-year follow-up study
Source: Sci Rep. 2020 Jun 15;10:9607. doi: 10.1038/s41598-020-66614-5 (PMC7296002; doi:10.1038/s41598-020-66614-5)
Supplement: Supplementary file 1 — Supplementary Information. [file 41598_2020_66614_MOESM1_ESM.docx]

**Risks factors and mortality of patients undergoing hip fracture surgery: a one-year follow-up study.**

Pierre Huette, Osama Abou-Arab, Az-Eddine Djebara, Benjamin Terrasi, Christophe Beyls, Pierre-Grégoire Guinot, Eric Havet, Hervé Dupont, Emmanuel Lorne, Alexandre Ntouba, Yazine Mahjoub^.^

**Supplementary file:**

**Table Demographic data according to time to surgery.** Data are expressed as median [interquartile space] or numbers (percentage). **BMI:** body mass index; **ASA:** American Status Anaesthetist.

| **Variables** | Time to surgery  *≤ 48 hours*  (n=128) | Time to surgery  *> 48 hours*  (n=181) | **p** |
| --- | --- | --- | --- |
| Age (years) | 82 [74-89] | 85 [78-89] | 0.054 |
| Male gender (n; %) | 36 (28) | 46 (25) | 0.595 |
| BMI (kg m^-2^) | 23 [20-27] | 24 [21-27] | 0.240 |
| Albumin level (g l^-1^)  <20  20-30  >30  Missing data | 6 (5)  44(34)  62(48)  16 (13) | 10 (5)  81(45)  67(37)  23 (13) | 0.316 |
| Comorbidities (n; %)  Diabetes  Hypertension  Dyslipidaemia | 19 (15)  82 (64)  32 (25) | 35 (19)  138 (76)  60 (33) | 0.309  0.020  0.123 |
| Residential status (n; %)  Own home  Nursing home | 91 (71)  37 (29) | 124 (69)  57 (32) | 0.627 |
| Pre-fracture functional status (n; %)  *Full independence*  *Partial dependence*  *Total dependence*  *Missing data* | 37 (29)  13 (10)  11 (9)  67 (52) | 47 (26)  24 (13)  28 (16)  82 (45) | 0.221 |
| ASA status (n; %)  1  2  3  4 | 6 (5)  49(38)  70 (55)  3 (2) | 3 (2)  42 (23)  122 (67)  14 (8) | 0.003 |
| Lee score (n; %)  0  1  2  3 | 82 (64)  30 (23)  13 (10)  3 (3) | 80 (44)  60 (33)  28 (16)  13 (7) | 0.005 |

**Table. Demographic data by cause of delay.** Datas are expressed as median [interquartile space] or numbers (percentage). **BMI:** body mass index; **ASA:** American Status Anaesthetist.

|  | Antithrombotic drug management  (n=39) | Operating ranges  (n=74) | Need for Additional test  (n=68) | p |
| --- | --- | --- | --- | --- |
| Age (years) | 86 [85-90] | 84 [71-89] | 86 [81-90] | 0.054 |
| Male gender (n; %) | 9 (28) | 17 (23) | 20 (29) | 0.879 |
| BMI (kg m^-2^) | 23 [21-30] | 24 [21-27] | 24 [21-26] | 0.240 |
| Albumin level (g l^-1^)  <20  20-30  >30  Missing data | 4 (10)  10 (26)  18 (46)  7 (18) | 3 (4)  38 (51)  27(37)  6(8) | 3 (4)  33(49)  22(32)  10 (15) | 0.298 |
| Comorbidities (n; %)  Diabetes  Hypertension  Dyslipidaemia | 11 (28)  35 (90)  18 (46) | 10 (14)  49 (66)  22 (30) | 14 (21)  54 (80)  20 (30) | 0.306  0.008  0.089 |
| Residential status (n; %)  Own home  Nursing home | 30 (77)  9 (23) | 50 (68)  24 (32) | 44 (65)  24 (35) | 0.536 |
| Pre-fracture functional status (n; %)  *Full independence*  *Partial dependence*  *Total dependence*  *Missing data* | 9 (23)  7 (18)  5(13)  18 (46) | 25 (34)  9 (12)  10 (14)  30 (40) | 13 (19)  8 (12)  13 (19)  34 (50) | 0.407 |
| ASA status (n; %)  1  2  3  4 | 0 (0)  7 (18)  26 (67)  6 (15) | 2 (3)  20 (27)  45 (61)  6 (8) | 0 (0)  15 (22)  51 (75)  2 (3) | 0.019 |
| Lee score (n; %)  0  1  2  3 | 9 (23)  21 (54)  7 (18)  2 (5) | 39 (53)  26 (35)  5 (7)  4 (5) | 32 (47)  13 (19)  16 (24)  7(10) | < 0.001 |

**Table. One-year mortality hazard ratio for LEE Score Criteria following surgical hip fracture management**. OR: Odd ratio; Binary logistic regression

|  | OR | CI 95% 0R | P value |
| --- | --- | --- | --- |
| Chronic kidney Disease  (Preoperative Serum creatinine > 177 μmol/L) | 1.646 | 0.343-7.895 | 0.533 |
| Congestive Heart Failure | 1.293 | 1.100-2.900 | < 0.001 |
| Diabetes on insulin | 1.223 | 0.512-2.919 | 0.651 |
| Coronary Artery Disease | 4.096 | 1.655-10.651 | 0.002 |
| Cerebrovascular Disease  (history of transient ischemic attack or stroke) | 1.884 | 0.858-4.135 | 0.114 |
